# Geological controls of mineralization occurrences in the Egyptian Eastern Desert using advanced integration of remote sensing and magnetic data

**DOI:** 10.1038/s41598-024-66924-y

**Published:** 2024-07-19

**Authors:** Ahmed M. Eldosouky, M. Eleraki, Aya Mansour, Saada A. Saada, Sara Zamzam

**Affiliations:** 1 Department of Geology, Faculty of Science, Suez University, P.O. Box 43221, Suez, Egypt; 2 Department of Geology, Faculty of Science, Zagazig University, P.O. Box 44519, Zagazig, Egypt

**Keywords:** Arabian–Nubian shield, Najd Fault System, Landsat-9, Magnetic data, Mineralization deposits, Eastern Desert, Solid Earth sciences, Geology, Geophysics

## Abstract

This study presents a comprehensive analysis of mineralization exploration in the Egyptian Eastern Desert (ED), one of the most sought-after areas for those interested in mining industry, by integrating Landsat-9 images and geophysical magnetic data. Employing advanced techniques like Principal Component (PC) analysis, Minimum Noise Fraction (MNf) transform, and Band-Ratio (B-Ratio), the research focuses on mapping lithological units, hydrothermal alteration regions, and structural elements. Composite images derived from specific PC, and MNf bands, and B-Ratio exhibit superior lithological unit identification. The findings emphasize that there are significant variations in the types of rocks extend from the southern to the northern parts of the ED. Hydrothermal alteration mapping, guided by B-Ratio results, aids qualitative lithological discrimination. A novel false color composite image optimizes Landsat-9 B-Ratios, enhancing rock unit discrimination. Correlation analyses reveal associations between mineralization types and major lithological units, while exploration of the magnetic anomaly map highlights its role in correlating mineralization sites. Structural features, analyzed through Center for Exploration-Targeting Grid-Analysis (CET-GA) and Center for Exploration-Targeting Porphyry-Analysis (CET-GA) with Tilt Derivative of RTP (TDR) techniques, contribute to a robust association between regions with medium to high structural density and porphyry intrusions and mineralization. The study significantly supports the advanced exploration geoscience, providing insights into the geological structures and dynamics governing mineralization in the Egyptian ED.

## Introduction

**Figure 1:**
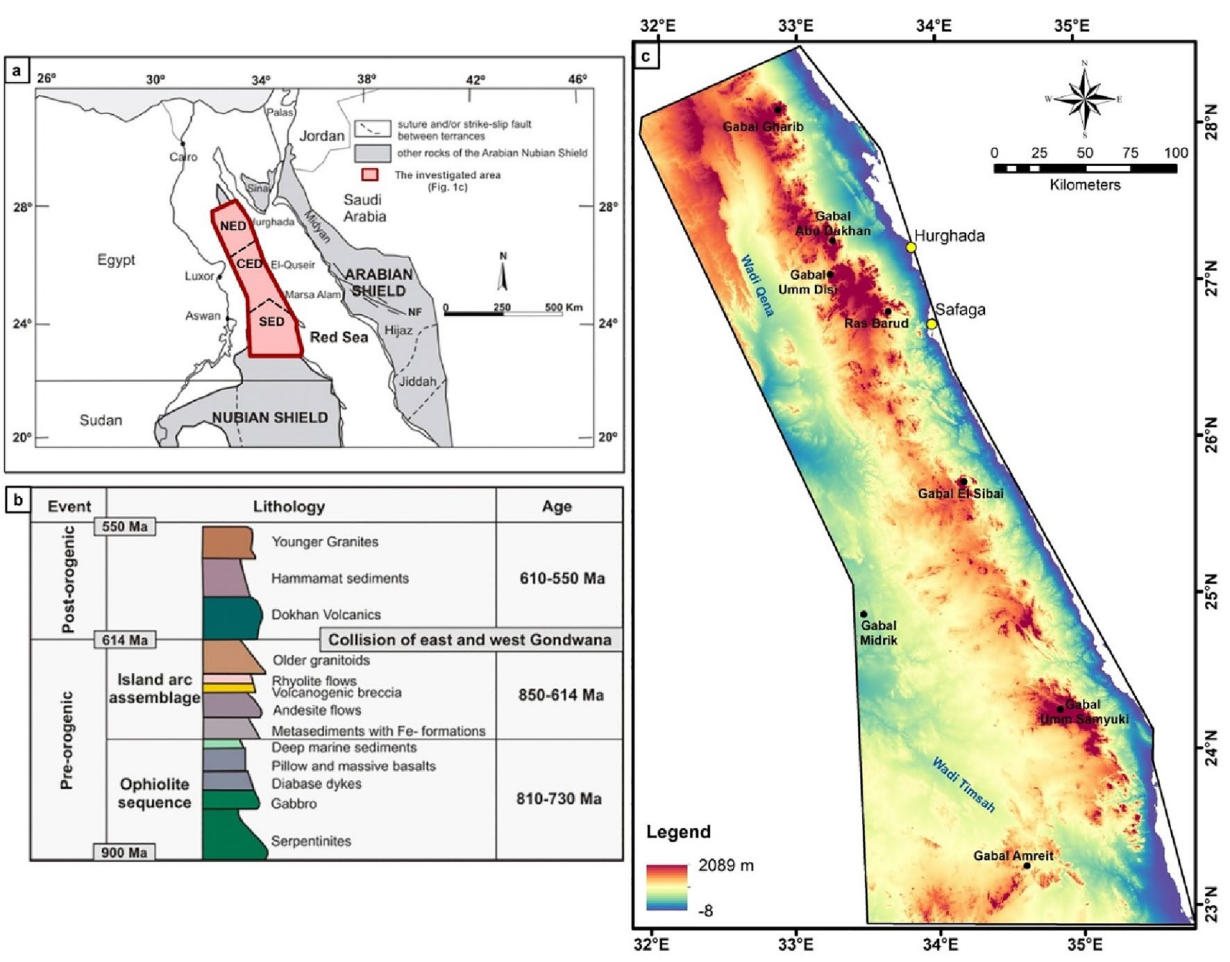
**(a)** Simplified map of the Arabian-Nubian Shield (ANS), after Johnson et al., 2011. (NED: North Eastern Desert, CED: Central Eastern Desert, SED: South Eastern Desert, NF; Najd Fault); (**b**) Lithostratigraphy with major tectonic events of the Pan-African basement complex in the ED (modified after Moghazi^48^); (**c**) Shuttle Radar Topography Mission Digital elevation map (SRTM DEM, Global 1 arc second—30 m resolution) of the interested area that downloaded from https://earthexplorer.usgs.gov/.

Notably, the Eastern Desert (ED) of Egypt is considered the main target for mineralization exploration and exploitation. It is affluent by metallic and non-metallic mineral resources approximately seventy minerals of economic quality, including gold, iron minerals, black sand, coal, manganese, sulfur, and other large economic raw materials. According to the latest statistics issued by the Egyptian Mineral Resources Authority (EMRA) regarding the amount of extracted minerals, such as an average of 6.7 million ounces of gold, and 50 million tons of coal, Egypt has the prospective to be one of the best mining locations in the world. Most of the ancient and newly agreed mining activities are located in the central and southern sectors of ED (Fig. 1a).

The geology of ED is very interesting as their exposed lithological units cover a wide range of geologic time from Precambrian to Recent. As a result of these rocks being affected by many tectonic events throughout geological history, this has led to a change in composition, mineralogy, and texture of rocks during hydrothermal processes associated with these events. These various alteration zones extend in vertical and lateral directions for kilometers, which it is the main destination for all those interested in mining in Egypt. Accordingly, systematic mapping of hydrothermal alteration haloes can help in indicating areas with potential for the discovery of mineral deposits. The petrology, ore mineralogy, radioactivity, geotectonic and structural settings of many locations in ED have been studied by various authors^1–7^. Despite these studies, there are many large areas that have not been studied before due to their strong rugged relief, extensive regolith cover and poor access conditions. On the other hand, and regardless of these challenges, these areas are considered targeted Greenfields.

Reliable geosciences information in the form of geological and mineralogical potential maps is very important for exploration and development of mineral resources. Mineral resource development has played an important role for sustainable economic growth. Conventional geological mapping and mineral exploration are labor intensive and require high investment, and take long periods of investigation, particularly for large areas. Contrary, modern exploration techniques are cost effective and can be achieved in less time and help to isolate potential areas from non-interesting areas for further exploration. The integration of satellite data analysis, and spaceborne magnetic interpretation are fast and economic methods that have been used in mineral exploration and hydrothermal zones detection^8–10^.

The most widely used satellite image in mineralization mapping is Landsat because it is multispectral image, characterized by high spatial resolution as well as downloaded it for free. Landsat sensor images from its various missions have been used successfully for discrimination between altered/unaltered rocks and hydrothermal alteration zonation in various regions^11–16^. Early and accurate up-to-date geological maps are essential guides in mineral potentiality delineation as it represents the most basic information for directing exploration activities^17–20^.

Space geophysical magnetic data have been extensively utilized for delineating structural features and mineralization zones, as illustrated by prior studies^21–30^. Magnetic anomaly maps, due to the heightened magnetic response of metamorphic rocks, offer important insights into the subsurface structure of metamorphic area^31^. The interpretation of these magnetic maps can furnish valuable information on inner structures potentially associated with magmatic feeding systems and linked to hydrothermally altered terranes^32^.

Understanding the geometry and distribution of subsurface features, such as folds, faults, fractures, and intrusions, is imperative for deciphering tectonic events, assessing mineral potential, and locating hydrocarbon reservoirs^26,33–38^. In recent decades, magnetic data has emerged as a valuable and cost-effective tool for investigating geologic structures, owing to its widespread availability, non-invasive nature, and sensitivity to magnetic anomalies associated with various subsurface features^39,40^.

The primary objective of a geophysical magnetic survey is to identify the subsurface geological setting based on variations in the Earth’s magnetic field, manifesting as anomalies due to the variations in magnetic susceptibility between the basement complex and the sedimentary cap^41^. The geophysical magnetic method finds diverse applications, including locating faults, folds, shear zones, contacts, and determining geological structures relevant to mineral exploration.

Integrating remote sensing and space geophysical magnetic data has been integral to accurately map lineaments. Through the utilization of remote sensing technologies, such as digital elevation models and Landsat-8 data, in conjunction with space geophysical magnetic data like airborne and satellite magnetic data, researchers can attain a comprehensive understanding of both surface and subsurface tectonic features. This advanced integrated approach has consistently proven to be a potent approach in geological exploration and analysis^21–23,42–47^.

**Figure 2:**
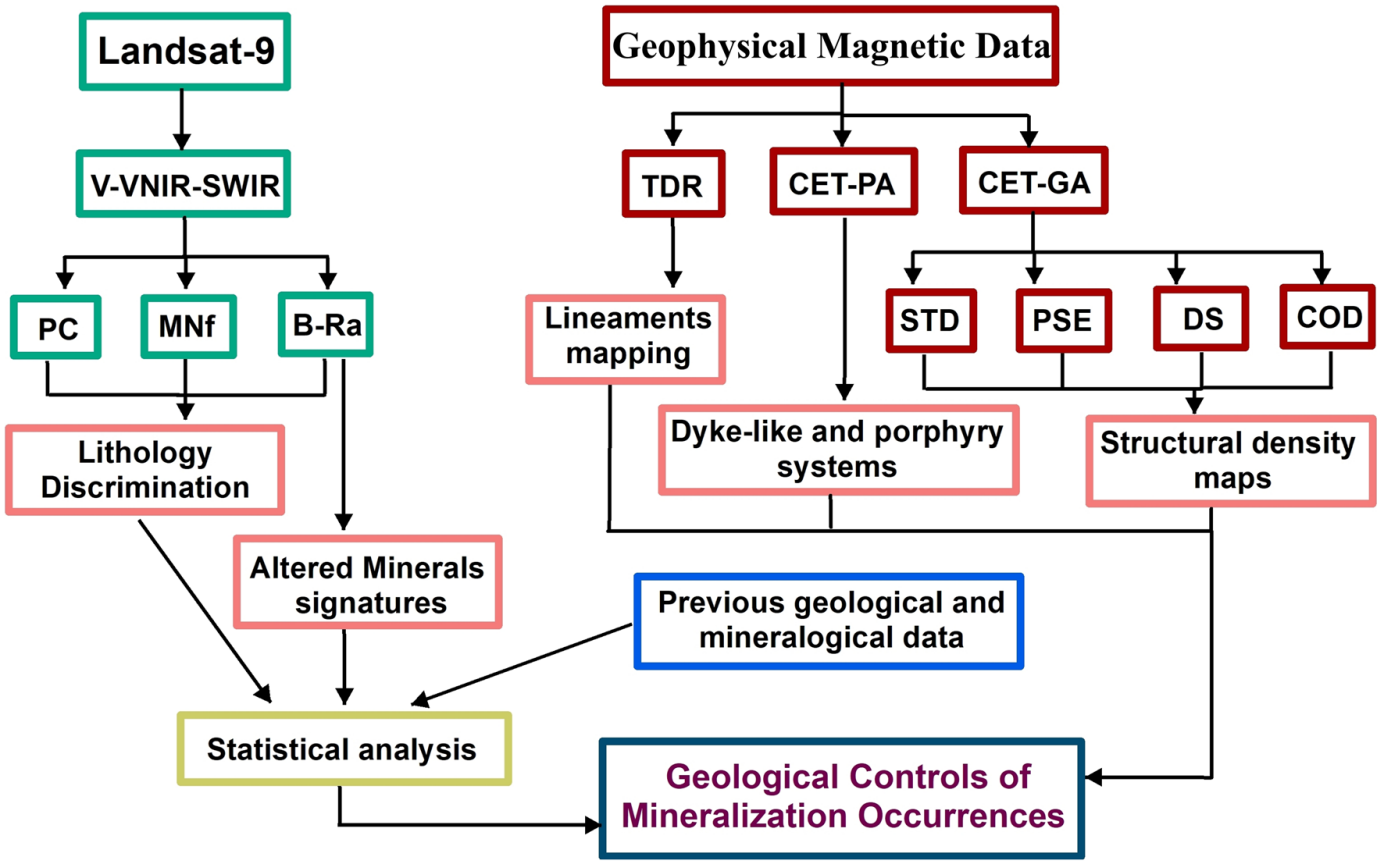
Methodology flowchart provides a clear organizational structure of the research study and layout results.

In general, the present assessment of the advantages of remote sensing and space geophysical magnetic datasets is to evaluate the predefined mineralization occurrences and to characterize the high possibility of new ones. The most important mineral deposits that occurred in ED are gold, iron, manganese, and phosphate deposits, but they are untapped mineral resources. This is due to the extreme complexity of the geography and geology of ED, which is considered the main reason for the slow progress of mineral exploration in it. Accordingly, the objectives of this investigation are (1) identifying the spatial distribution of various mineralization zones in the investigate area based on the interpretation of Landsat-9 images; (2) retrieving information about the subsurface geology based on the analysis and interpretation of space geophysical magnetic data, integrated with available geological information; (3) evaluating the control of geological features on mineralization zones; and (4) constructing a model that can be used as a guide for further detailed follow-up mineral exploration works in the area (Fig. 2). Our study integrates space geophysical magnetic data and Landsat-9 images, utilizing advanced techniques such as Band-Ratio (B-Ratio), Minimum Noise Fraction (MNf) transform, and Principal Component Analysis (PCA). The application and integration of these advanced approaches in mineral exploration of the Eastern Desert (ED) will produce a substantial advancement. Our study presents valuable assistance to mineral exploration by handling the distinguishing challenges of the ED and advancing our knowledge of mineralization processes in this complicated geologic setting.

## Geological framework and mineralogical data

Egypt's Precambrian rock complexes cover approximately 10% of the country's total area. It is mainly found in the Eastern Desert (ED) along the Red Sea (Fig. 1a). A significant portion also includes southern Sinai. In the ED, the bedrock extends in a belt over approximately 800 km. These rocks are unconformably overlain on their western and eastern margins by Nubian Sandstone, Miocene, and younger sediments^49^. The basement rocks are associated with the Arabian–Nubian Shield, where mafic/ultramafic magma assemblages were formed in a plate subduction environment consisting of oceanic crust and immature island arc material. On the other hand, calc-alkaline volcanisms are remnants of an overripe island arc complex. The final accretion of the various island arcs caused strong tectonic deformation during the so-called Pan-African orogeny (Fig. 1b). Granitic intrusions and felsic volcanics (post-formation) are thought to represent the collision of this island arc with the African craton^50^.

**Figure 3:**
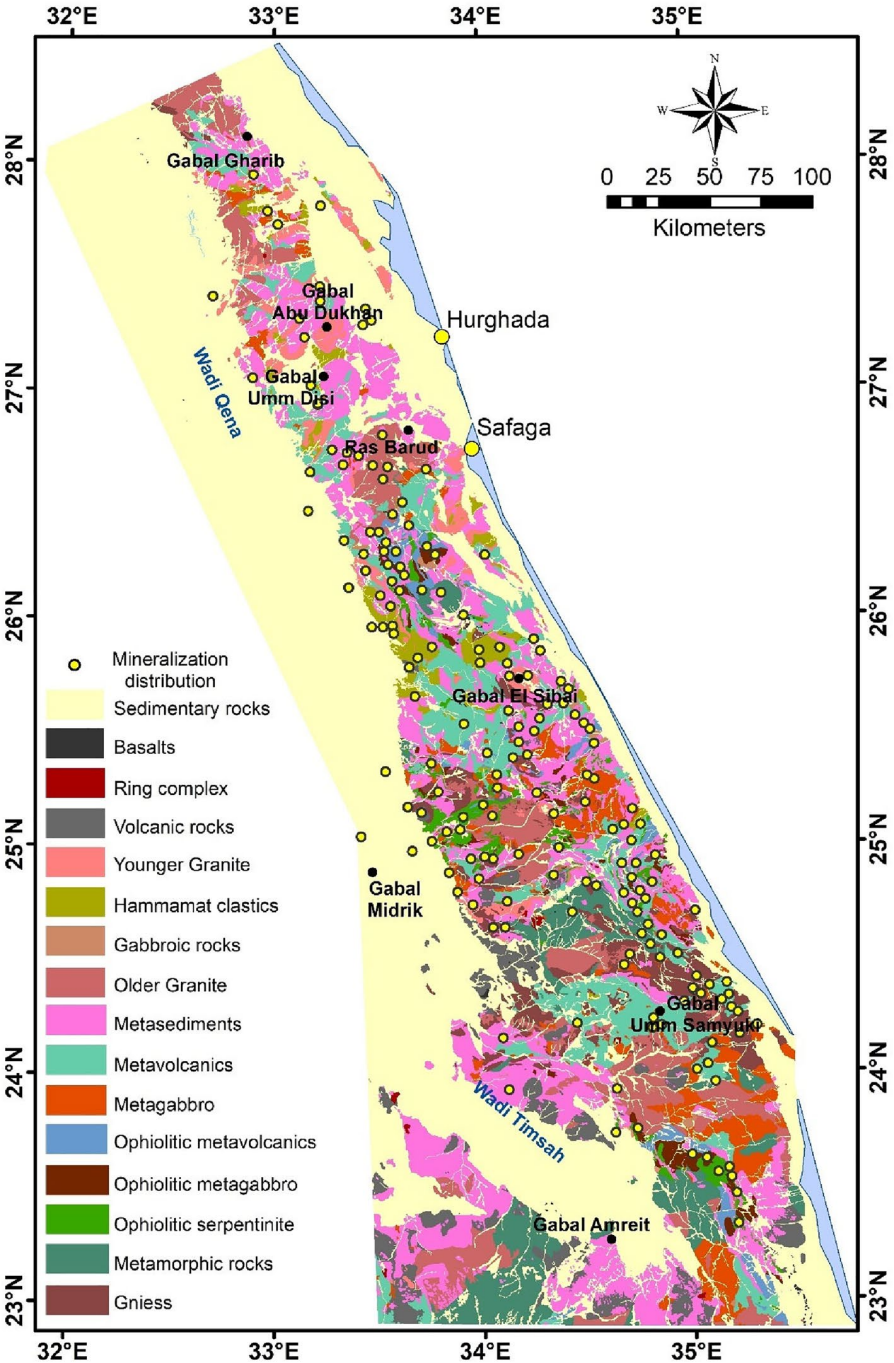
Geological map of the investigated area (modified after Conoco^71^) (By ArcGIS v.10.3. https://www.esri.com/en-us/arcgis/products/arcgis-desktop/overview/).

In general, ED land is divided into northern, central, and southern regions based on the significant proportion and age determination of gneisses, granites, and ophiolites^51,52^. The basement complex contains four main litho-tectonic units (Figs. 1b, 3): (a) gneisses and migmatites, (b) ophiolites with island-arc assemblages, (c) syn to late post-tectonic granites, and (d) Dohan volcanoes with Hamamat molasses deposits^53^. Ries et al.^54^ considered the sequence of rocks within the ED to be a tectonic sequence rather than a stratigraphic one. Authors believe that these domains are juxtaposed along major shear zones, with the Qena-Safaga shear zone detached between the NED and CED, as well as the Idfu-Mersa Alam shear zone divided the CED from SED^55,56^.

Furthermore, the tectonic history of the ANS comprises seven phases: (1) division of Rodinia (~ 850 Ma); (2) seafloor extension to form oceanic lithosphere (~ 750 Ma); (3) subduction and creation of island arc volcano-sedimentary sequences (~ 720 Ma); (4) accretion and impact of the island arc series; (5) intrusion of older granites (~ 610 Ma); (6) disinterment and orogenic collapse (~ 570 Ma); and (7) invasion of alkalic, post-orogenic younger granites (~ 474 Ma)^56–58^. In general, four main faulting were differentiated within area include NW–SE; NE–SW, N–S and E–W directions. Some of them are very deep-seated and characterized by a multi-phased development as well as great spatial extension^6^.

**Figure 4:**
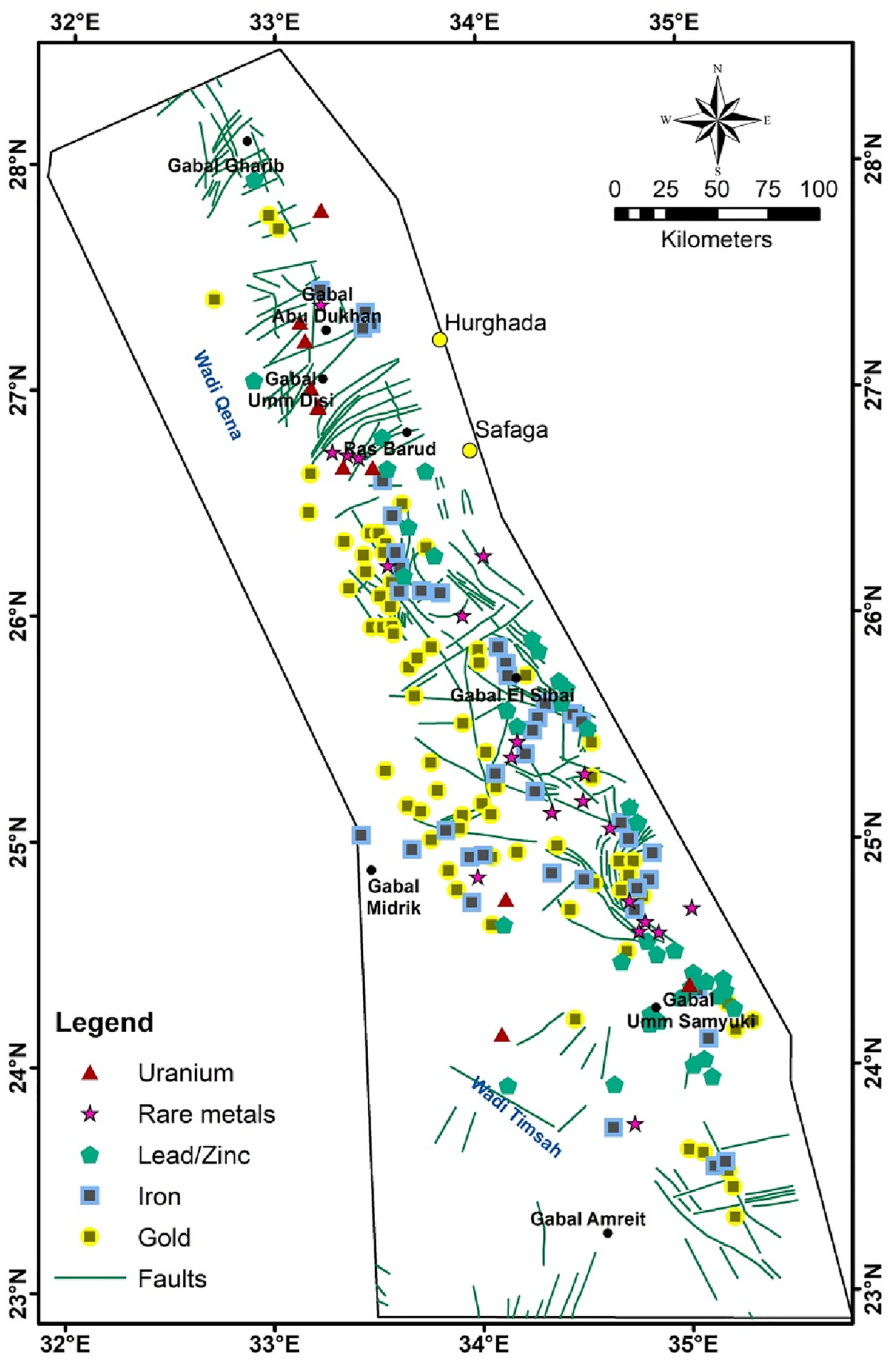
Mineralization distribution and structural map of the interested area (redrawn after Egyptian Geological Survey and Mining Authority EGSMA^72^).

The investigated area is characterized by a lot of mineralization in various mode of occurrence, nature of mineralization and places of economic potential hosted by the Pan-African Late Proterozoic basement rocks (e.g., gold, iron, lead, rare metals, radioactive minerals, …etc.; Fig. 4). It is noticeable that there is a comparison between the places of mineralization with high fracture density throughout terranes and geological time. Large zones of alteration have materialized with hydrothermal fluid flow during fault/fracture network development. The most explored and widespread metallic minerals in the region are gold and iron deposits (Fig. 4). Historically, most known gold deposits were discovered and exploited by the ancient Egyptians using primitive techniques^59,60^. Currently, mineral exploration relies on a variety of exploration techniques, including geochemistry, geophysics, geological mapping, aerial photo interpretation, and ground surveying. On the other hand, there is a degree of uncertainty and mineral exploration risk, especially when using a single approach. Therefore, it is now common to use multiple techniques for mineral redemption when possible^9^.

Generally, three main stages of gold mineralization are recognized in the Egyptian ED^61^. It was suggested that gold deposits are controlled by structures. The initial stage is reflected by gold-bearing quartz veins confined to brittle-ductile shear zones genetically related to island-arc accretionary tectonics. In most cases, mineralized veins are accompanied by alteration zones of gold-bearing wall rocks, and the alteration pattern includes sulphidization, silicification, and carbonation. The initial stage of gold mineralization is mainly concentrated in the southern part of the Egyptian ED. The second stage of gold mineralization^61^ is mirrored in gold-bearing quartz and carbonate veins developed along shear and tensile fractures associated with back-arc basin closure tectonics in the CED. Meanwhile, the third stage of gold mineralization is reflected in barite veins and gold-copper-bearing quartz veins associated with gossan. Mineralization is confined to shear and tensile fracture systems formed in the Dokhan volcanoes and their granitic equivalents in the northern ED intrusions of Egypt. This mineralization may be related to the emplacement of younger granitic intrusions and recent continental east–west impact tectonics that affected this area during early Arabian–Nubian shield evolution^62^.

On the other hand, the Egyptian iron minerals are of strata bound type, which ranging in age from Proterozoic to Cenozoic^63^. These minerals show diverse origins, geological conditions and formations, as well as restricted to the CED. Particularly, banded Iron Formations (BIF) are typically associated with the Precambrian era and are characterized by alternating layers of iron-rich minerals (volcanic-volcaniclastic successions) of usually andesitic composition. These BIF successions are confined to Pan-African (Proterozoic) thrusts, overlain by ophiolitic mélange complexes, and occur in discrete locations in the CED (Fig. 4). Some of BIF successions are related to early Proterozoic such as the Umm Nar deposit^64^, while other occurrences are probably of late Proterozoic volcanic origin in island-arc environments^65^.

Contrary, uranium, lead/zinc, and rare metals are the least widespread in the ED and have been rarely studied. The few locations that have been explored are located at many places of the NED, CED, and SED. The main types of uranium deposits in the ED can be classified as follows according to the IAEA classification^65^: i-Metamorphic deposits (Metamorphosed Sandstone) and ii-vein types such as at Gabal Gattar, Um Samra-/Um Bakra, and El-Sella granite. Genetically, the mineralization of rare metals is associated with the final stage of intrusion of granites formed either by magma, post-magma, or Metasomatism by hydrothermal processes^66–69^. Lead/zinc ores occur in Egypt as replacement deposits in calcareous sandstone and limestone such as at Um-Gheig area, or as hydrothermal veins for metamorphic rocks such as at Um-Samiuki area, or as placer deposits in several areas^70^. Nuclear Materials Authority (NMA) in Egypt started an aspiring program to re-evaluate the basement rocks and to delineate the suitable conditions of these uncommon minerals that can compare with others in the world.

## Space-based datasets and methodology

### Remote sensed dataset

Cloud-free multi-sensor Landsat-9 products were utilized in the present study for hydrothermal mineralization and lithological mapping. Eleven Landsat-9 OLI/TIRS images were downloaded from the U.S. Geological Survey Earth Resources Observation and Science Center (EROS) (https://earthexplorer.usgs.gov/) to cover the interested area; their characteristics are listed in Table 1. We were careful to choose a small temporal resolution (time difference) between the utilized images in order to ensure spectral intimacy (radiance) between them, facilitate the analysis process, and accuracy of the results. Landsat-9 OLI/TIRS bands cover seven parts of the visible-near infrared (VNIR) and shortwave infrared (SWIR) spectrums (1–7 9 bands) with spatial resolution up to 30 m and two parts of the thermal infrared (TIR) spectrums (10–11 bands) with spatial resolution up to 100 m. on zone 36 North Universal Transverse Mercator (UTM) using the WGS-84 datum.

**Table 1:** Scene IDs of Landsat-9 OLI/TIRS imagery used in the investigated area.

Scene ID	Acquisition date
LC09_L2SP_174044_20230910_20230912_02_T1_SR	10/Sep/2023
LC09_L2SP_175040_20230917_20230919_02_T1_SR	17/Sep/2023
LC09_L2SP_175041_20230917_20230919_02_T1_SR	
LC09_L2SP_175042_20230917_20230919_02_T1_SR	
LC09_L2SP_176040_20230924_20230926_02_T1_SR	24/Sep/2023
LC09_L2SP_174041_20230926_20230928_02_T1_SR	26/Sep/2023
LC09_L2SP_174042_20230926_20230928_02_T1_SR	
LC09_L2SP_174043_20230926_20230928_02_T1_SR	
LC09_L2SP_173042_20231005_20231006_02_T1_SR	05/Oct/2023
LC09_L2SP_173043_20231005_20231006_02_T1_SR	
LC09_L2SP_173044_20231005_20231006_02_T1_SR	

The downloaded satellite images (collection 2 level 2) include several radiometric and atmospheric calibrations to enhance the quality of raw data and prepare them for analysis and processing techniques. The utilized images are on zone 36 North Universal Transverse Mercator (UTM) using the WGS-84 datum. The VNIR-SWIR bands of these images were stacked using ArcGIS packages, version 10.3 (developed by esri.com, https://www.esri.com/en-us/arcgis/products/arcgis-desktop/overview/), mosaicked and clipped to the interested area through QGIS, version (3.30) software (https://www.qgis.org/en/site/). The resultant full image was utilized for extracting the alteration mineral indices and distinguishing the lithological units through Band-Ratio (B-Ratio), Principle component (PC) and Minimum Noise fraction (MNf) analysis methods. Additionally, the mineralization signatures were extracted as density slices from B-Ratio results using their mean (M) and times the standard deviation (second or third SD) values^73^ to ensure accuracy (95% and or 98%) in mineralization mapping. Moreover, the pre-defined mineralization occurrences were correlated with different exposed geological units to evaluate and analyze their interrelationship. The processing and analysis methods were made by ENVI, version 5.1 (generated by L3Harris.com, https://www.l3harrisgeospatial.com/Software-Technology/ENVI) and the final images were exported using ArcGIS packages (https://www.esri.com/en-us/arcgis/products/arcgis-desktop/overview/).

Band ratio (B-Ratio) images were created simply by dividing the DN value of each pixel in one band by the value that corresponds to it in another band^74^. This technique is very effective and preferable for emphasizing specific features and clarity it more than the surrounding materials^15^. According to the purpose of this study, the various alteration types of the known mining locations (based on their different formational factors) and previous geological studies on many scattered areas as parts of the current study area, iron oxides, ferrous silicates, and clay/OH-group altered minerals were determined from Landsat-9 based on their spectral characteristics of altered minerals. In general, these altered minerals give an impression and indicate the various types of alteration zones as iron oxides are related to the oxidized zone; clay/OH-group minerals (kaolinite and calcite) associated with the propylitic zone; the diverse ferrous silicates minerals (chlorite) represented the broad alteration zones^71^. The iron oxides zone has the wider spectral absorptions that extend between 0.9 and 1.1 µm. On the other hand, minerals accompanied the propylitic zone have absorption characters at 2.1–2.5 µm due to fusions of the basic fluctuations and overtones, while their typical spectral reflectance recorded at 1.55–1.75 µm^75–78^. Consequently, multiple ratios between 2, 4, 6, and 7 bands (VNIR and SWIR regions^79^) were utilized for detecting the preferable mapping ratios of the altered minerals that based on their spectral curves from USGS spectral library.

PC analysis was computed on the Landsat-9 VNIR-SWIR input bands to differentiate the main exposed geological units. This method is a multivariate statistic that selects eigenvector loadings of variables (uncorrelated linear combinations) such that each component sequentially extracts a linear combination and has a lesser variance^80^. Moreover, the MNf transform is a discrete linear transform that includes two distinct PC analysis rotations and whitening steps to reduce noise levels. This step is helpful to define the rich intrinsic dimensionality and separate the noise components from the true signal^81^.

### Geophysical magnetic dataset

**Figure 5:**
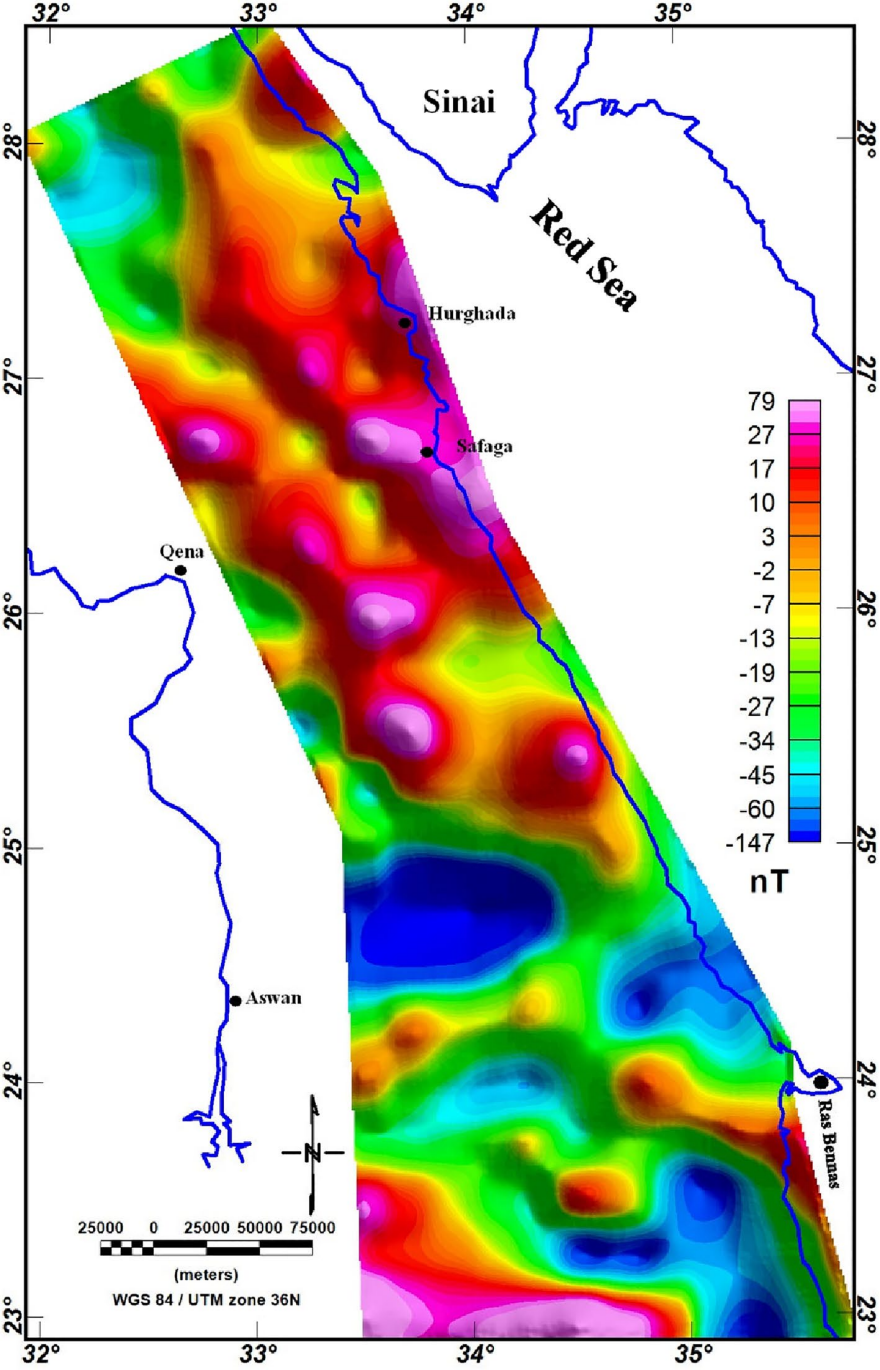
EMAG2 v3-magnetic anomaly map of the Egyptian ED (https://www.ncei.noaa.gov/products/earth-magnetic-model-anomaly-grid-2).

Magnetic data (Fig. 5) were acquired from the EMAG2 v3 (Earth Magnetic Anomaly Grid, https://www.ncei.noaa.gov/products/earth-magnetic-model-anomaly-grid-2) 2-arc-minute resolution dataset, curated by the Cooperative Institute for Research in Environmental Sciences (CIRES), utilizing a combination of airborne, marine, and satellite magnetic data^82^. The longer wavelength magnetic data (> 300 km) were sourced from the low-Earth orbit satellite CHAMP to generate the MF6 model, featuring a length-scale resolution of 333 km^82^. Short-wavelength magnetic data were incorporated wherever marine and airborne data were accessible. In this study, we utilized the comprehensive CIRES-compiled dataset, encompassing all available wavelength data for analysis. Magnetic data analysis is performed using Geosoft Oasis Montaj software (https://www.seequent.com/products-solutions/geosoft-oasis-montaj/).

#### The tilt derivative (TDR)

The Time-Domain Tilt Derivative (TDR) is employed on space geophysical magnetic data for deciphering shallow basement structures, exploring mineral areas, and delineating the edges of causative bodies^83^. The tilt filter, proposed by Miller and Singh^84^ and Verduzco et al.^85^, has been further developed by various researchers, including Salem et al.^86,87^ and Fairhead et al.^88^, gaining important attention for its essential and applied simplicity^89^. The TDR is determined by taking the arctangent of the vertical to the horizontal derivatives.

1 $$T{\text{DR = tan}}^{{ - 1}} \left( {VDR/THDR} \right)$$

Here, VDR represents the vertical derivative, and THDR stands for the total horizontal derivatives. In mathematical terms, the TDR is expressed as:

2 $$i.e.:\;T{\text{DR = tan}}^{{ - 1}} \left( {\frac{\partial f/\partial z}{{\sqrt {\left( {\partial f/\partial x} \right)^{2} + \left( {\partial f/\partial y} \right)^{2} } }}} \right)$$

Here, *f* denotes the magnetic or gravity field, while δƒ/δx, δƒ/δy and δƒ/δz represent the first derivatives of the field *f* in the x, y, and z directions, respectively. This methodology holds promise for its efficacy in analyzing potential field data and has proven relevance for applications in various geological regions.

#### Center for exploration-targeting grid-analysis (CET-GA) technique

The CET-GA Grid Analysis extension incorporates tools for Texture Analysis, Lineation Detection, Lineation Vectorization, and Structural Complexity to map lineaments and identify favorable regions for ore deposits^21,22,90–92^. This procedure strengthens discontinuity zones within potential field data, emphasizing variations in magnetic intensity. Structures are discerned in the data by distinguishing complex texture zones in the local magnetic response before searching for axes of symmetry. These axes typically represent linear disruptions in the signal, commonly linked to magnetic discontinuity zones due to rock edges, linear structures, and intrusions for understanding the geological setting of an area^21,22,93^.

Zones of magnetic discontinuity are mapped as skeletal structures through texture enhancement. The output data shows each region of the discontinuity zones as skeletal line segments, revealing variations in directions and offsets within the structural features^21,22,94^. This technique involves the following steps:

i. Texture Enhancement: Locates areas with complex textures associated with magnetic discontinuities.
ii. Phase Symmetry: Uses the results which are obtained from texture enhancement to delineate zones of lateral discontinuity.
iii. Delineation of Structures: Uses the results which are obtained phase symmetry to transform discontinuity-containing zones into line-like structures.

#### Center for exploration-targeting porphyry-analysis (CET-PA) technique

The CET Porphyry Technique initiated the implementation of the Circular Feature Transform (CFT)^95^, designed for mapping circular-shaped features. Subsequently, the centers of elevated or depressed circular features are detected. Using the Amplitude Contrast Transform (ACT), a circular feature manifests as a 'halo,' coinciding with the circular rim of the feature. The outputs include a database file specifying circular feature centers, radial symmetry strength, and the radius that gives the highest response (in both cells and meters). The polygon file encompasses, for each feature position, the circle boundary generating the strongest radial symmetry response, allowing visualizing the extent of detected circular features^21,22,96^.

## Results

The integration of results from the synthesis, analysis, and interpretation of various datasets including Landsat-9 images and space geophysical magnetic data is valuable in defining three essential factors in exploring potential mineralization. These mapped elements are lithological units, hydrothermal alteration regions, and structures. In lithological mapping studies, the more analysis methods are used, the more accurate and best geological results are generated.

**Figure 6:**
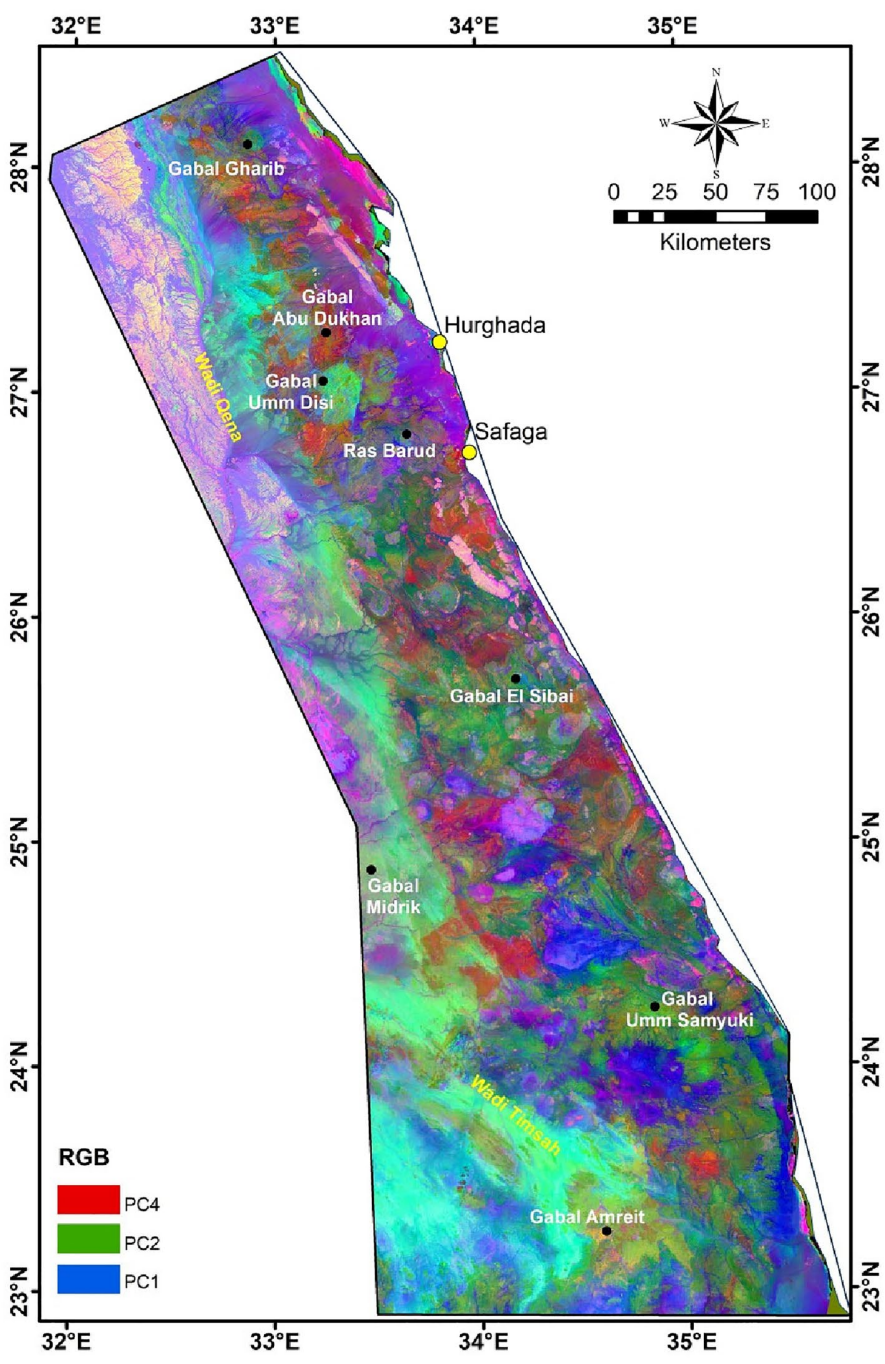
RGB color composite of Landsat-9 PC4, PC2 and PC1 images for the interested area.

**Figure 7:**
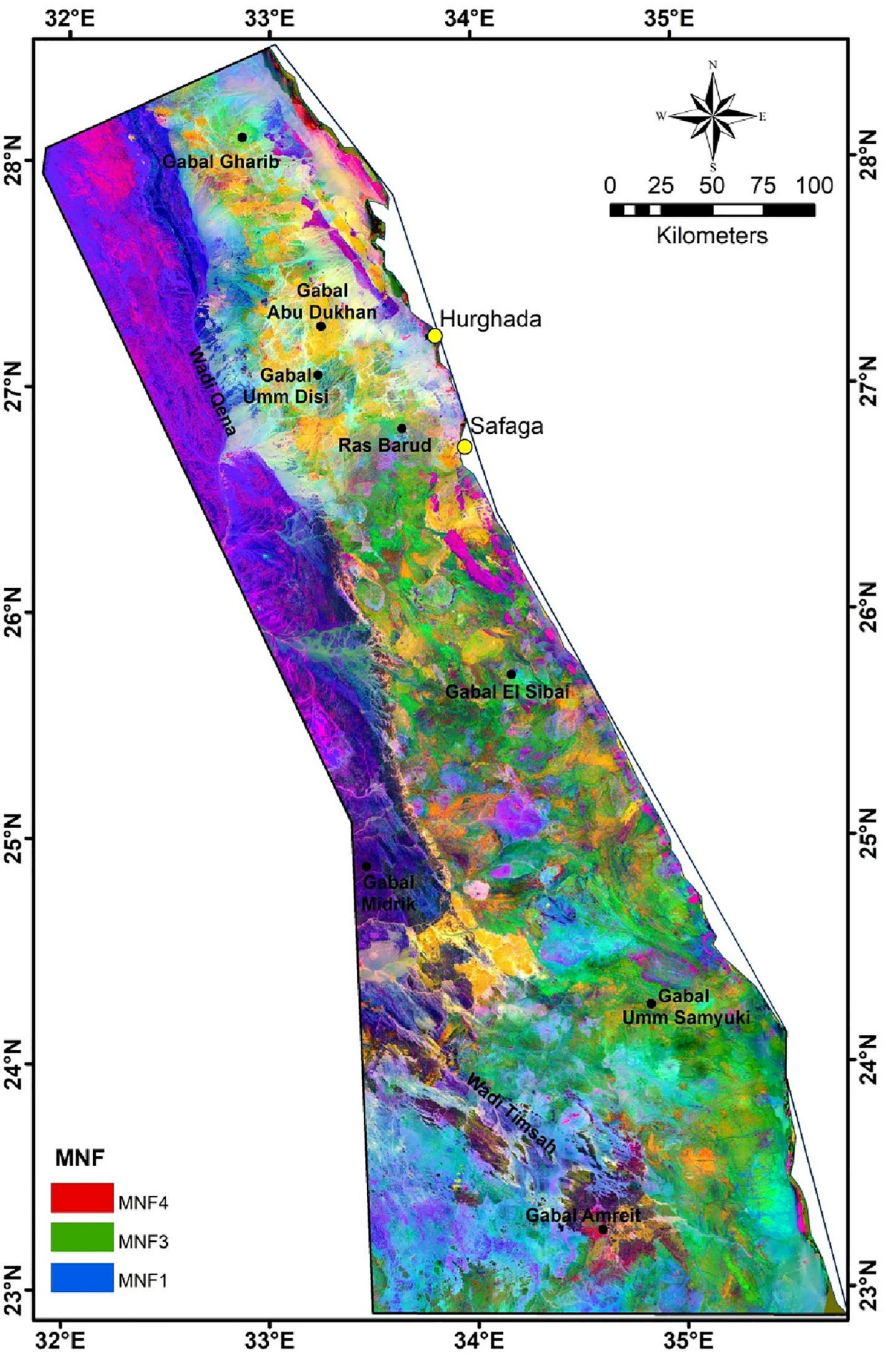
RGB color composite of Landsat-9 MNf4, MNf3 and MNf1 images for the interested area.

In this study, PC analysis and MNf transform are applied to facilitate the separation between diverse exposed rock units (Figs. 6, 7). The boundaries between different main rock assemblages in this area can be easily defined in the PC analyzed and MNf images. The composite images produced from (R: PC4, G: PC2, B: PC1) and (R: MNf4, G: MNf3, B: MNf1) for Landsat-9 give better results for lithological identification than other PCs and MNfs composite images (Figs. 6, 7). In addition, the results extracted from these two images complement each other, as each of them explains and shows some rock units that are different from the others. PC image highlights the older granites, metavolcanics/metasediments and metamorphic rocks in pale purple with blue, yellowish green, and dark green, respectively. On the other hand, MNf image displays the younger granite, ophiolite sequence, and sedimentary rocks as yellowish orange, shadows of brown, and light purple to dark blue colors, respectively.

There was difficulty in discriminating the boundaries of the Hammamat clastics in both images, as they appear in different shades of color, like most other rocks, particularly granitic ones. This may be due to their small spatial distribution and/or the relative closeness of their composition to other lithological units. This convergence is due to its origin, as it was formed during initial stage of N–S extension orogeny in the Neoproterozoic time, which sometimes leads to it being affected by intrusions from younger granites. This result is consistent with previous geological studies that focused on investigating Hammamat sediments in ED^97,98^. However, sometimes precise topography was a guide in distinguishing between the geological units, especially granitic rocks (Figs. 1c, 3).

It is evident from PC analyzed and MNf results that ophiolite sequence and metamorphic rocks are found in abundance in SED, while granitic rocks and Hammamat clastics are widespread in NED (Figs. 5, 7). In addition, the metavolcanics/metasediments rocks are the most abundant Precambrian rocks in ED, as they occurred in large amounts in the CED and SED. The results of the utilized analysis methods showed their effectiveness in classifying the basement rocks exposed in ED, despite the large extension of the investigated area, regarding their compatibility with the common lithological classification of ED into three sectors^51,52,99^. This diversity in the presence, origin, and tectonic events of the basement rocks is the main reason for the variety of mineral occurrences in the interested area.

**Figure 8:**
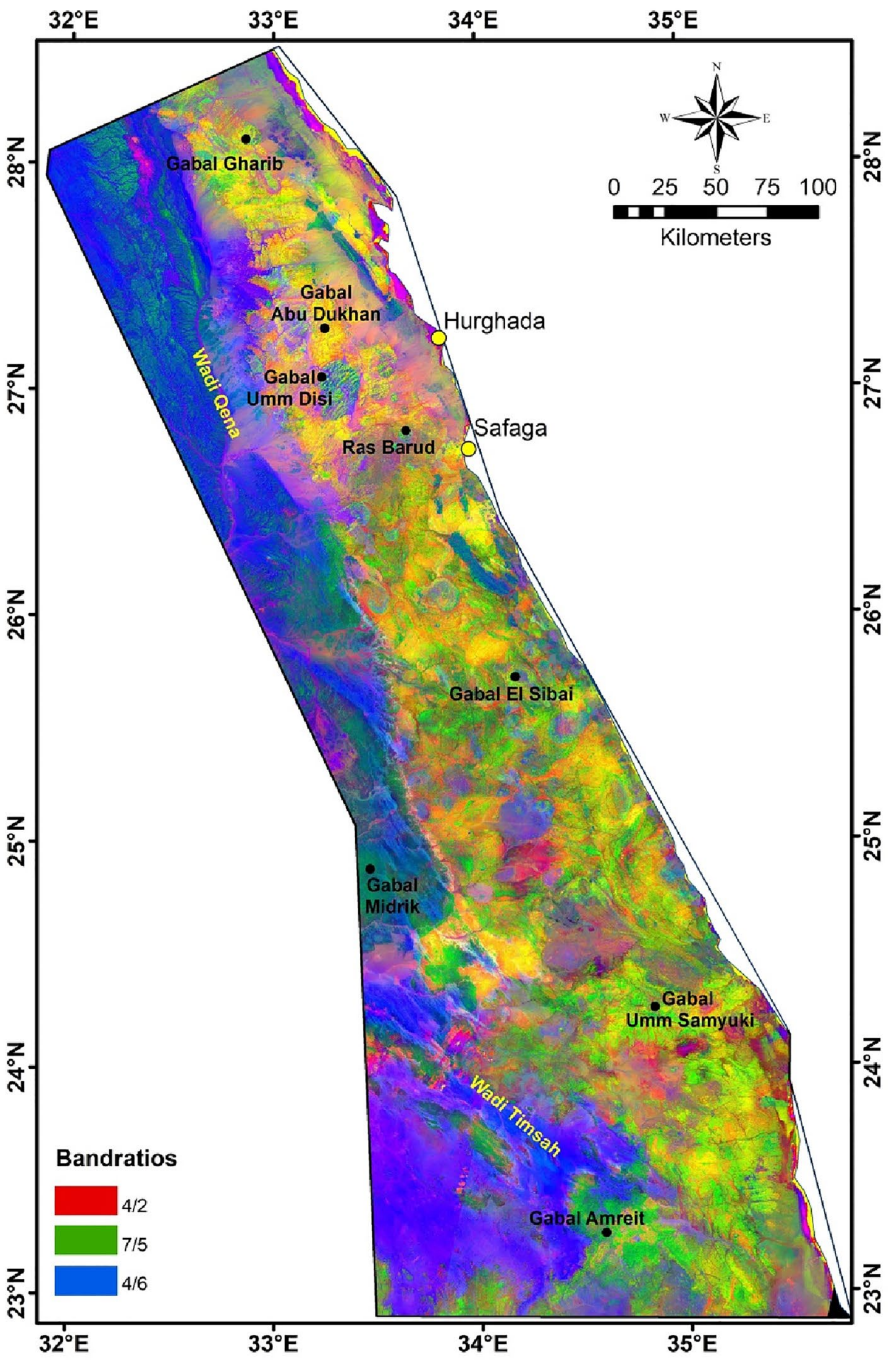
False color composite image of Landsat-9 B-Ratio (R: 4/2, G: 7/5, B: 4/6).

Logical mapping of hydrothermal alteration processes can help identify preliminary areas for various minerals discovery, which exist as a result or in association with these hydrothermal fluids. In our investigated area, B-Ratio results were used to discriminate the lithological units qualitatively as well as for detection of hydrothermally altered zones. False color composite (FCC) image, with Landsat-9 B-Ratio (R: 4/2, G: 7/5, B: 4/6) is the best ratio, clearly distinguishes rock units compared to the previous geological map (Fig. 8). These ratios are utilized based on several previous studies that focused on mapping rock units in various areas in ED^100,101^. The FCC image was enhanced by a nonlinear stretching technique (histogram equalization) in order to improve the contrast between the interested features. Granitic rocks are highlighted in shadows of red, metavolcanics/metasediments in green, metamorphic rocks in blue, sedimentary units in greenish blue, and ophiolite sequence in yellowish orange (Fig. 8). Moreover, B-Ratio of altered minerals include 4/2; 7/6; 6/7 are utilized for extracting and tracing iron oxides, ferrous silicates, and clay/OH group minerals, respectively. Each ratio represents a specific zone of ​​these altered areas in order to give a general overview of the distribution of these minerals and the majority of the altered mineral signatures in the area (Fig. 8).

**Figure 9:**
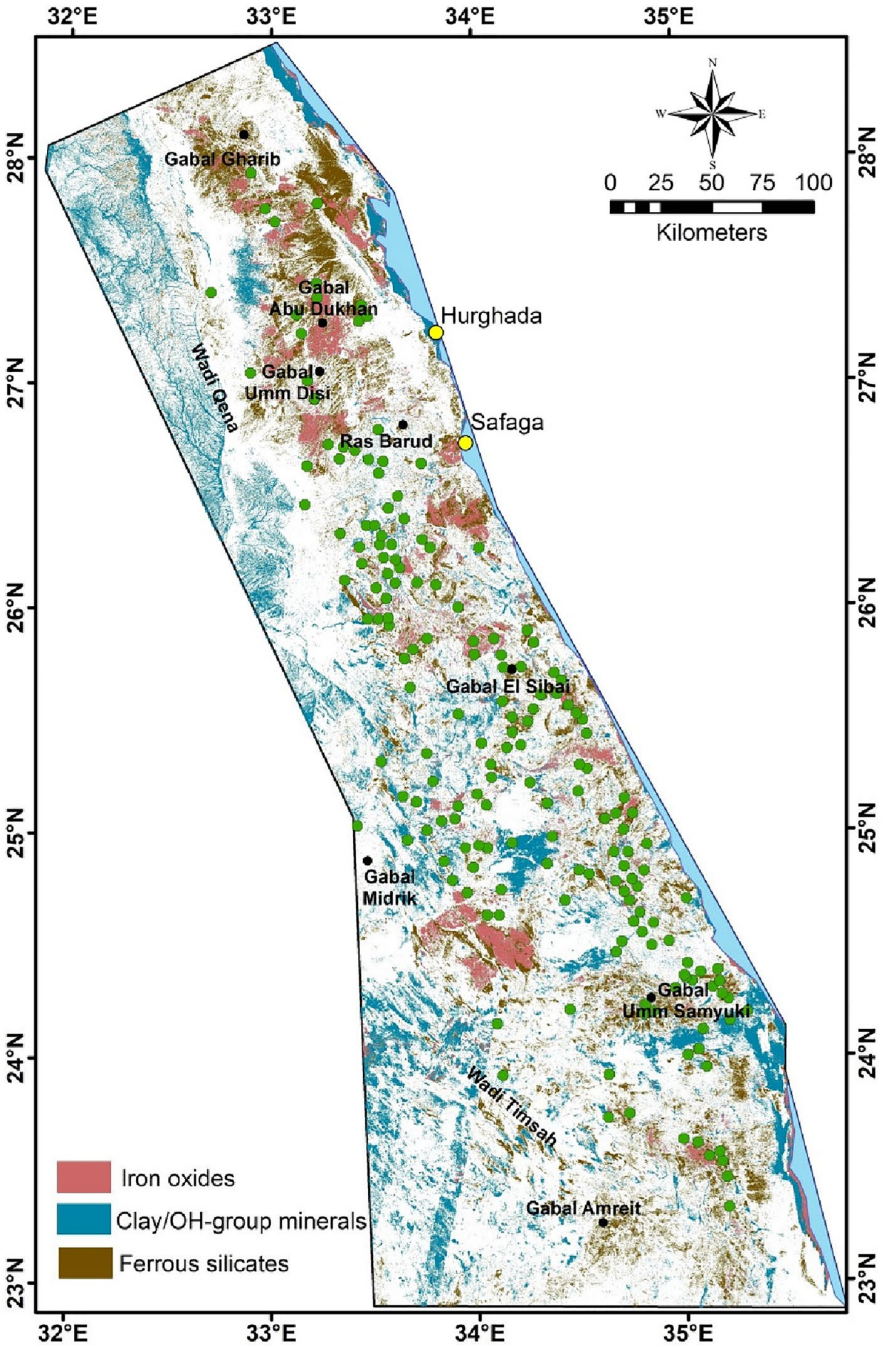
Alteration mineralogical signatures extracted from B-Ratio images for the interested area; green circles related to known mineralization localities, see Fig. 3.

It is observed from the distribution of these mineral signatures that they are not related to weathering processes, but rather indicate specifically and accurately the areas of alteration and mineralization, particularly iron oxides (98% accuracy value) and clay/OH group minerals (95% accuracy value; Fig. 9). While ferrous silicates minerals (95% accuracy value) appear in abundance in NED, it may be due to the presence of differentiated groups (I-III) of younger granite (for example, group I have been extensively silicified), Hammamat clastics, and intermediate to felsic volcanism^102,103^.

**Figure 10:**
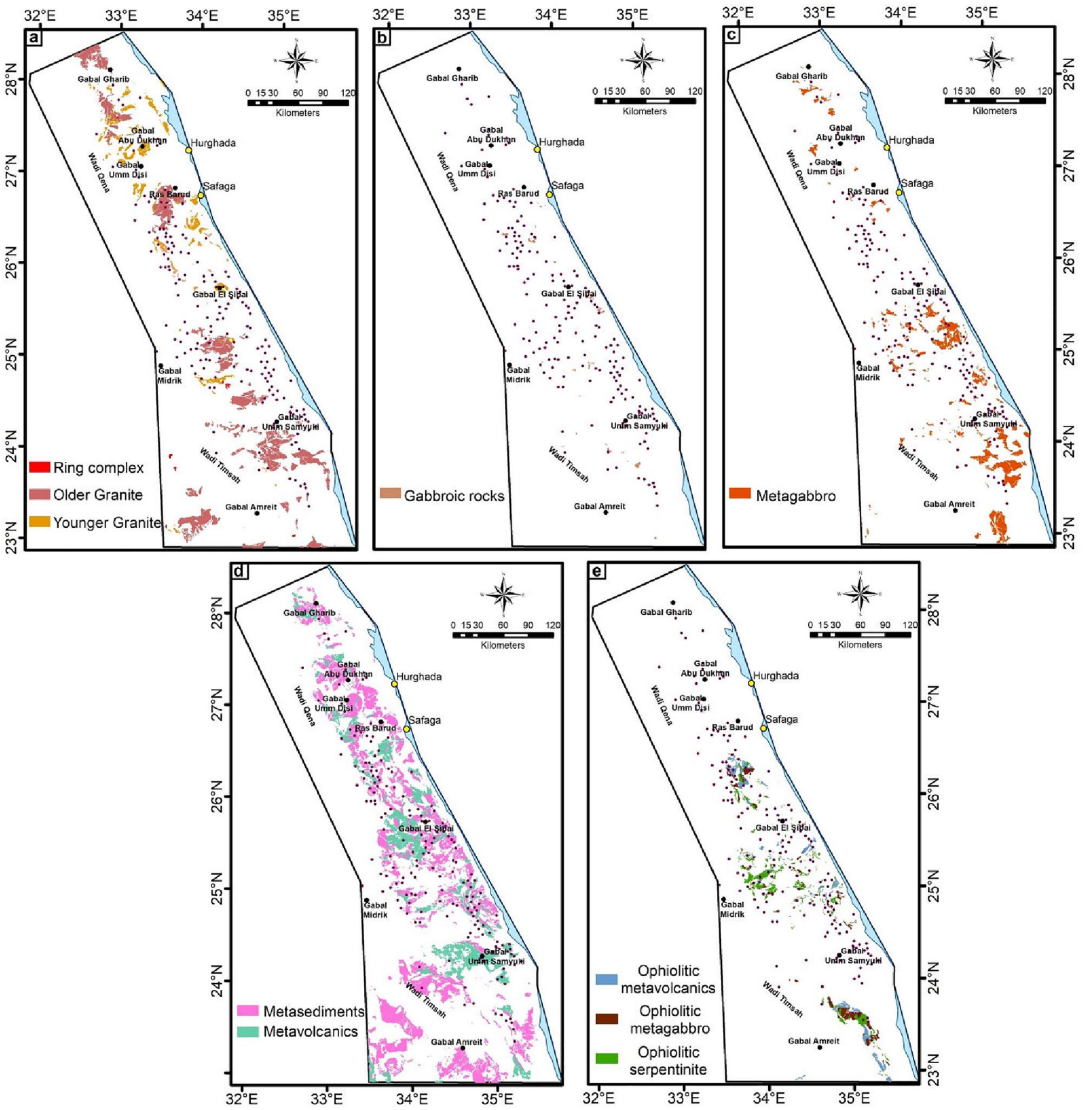
Distribution of Mineralization localities related to various lithological units.

**Figure 11:**
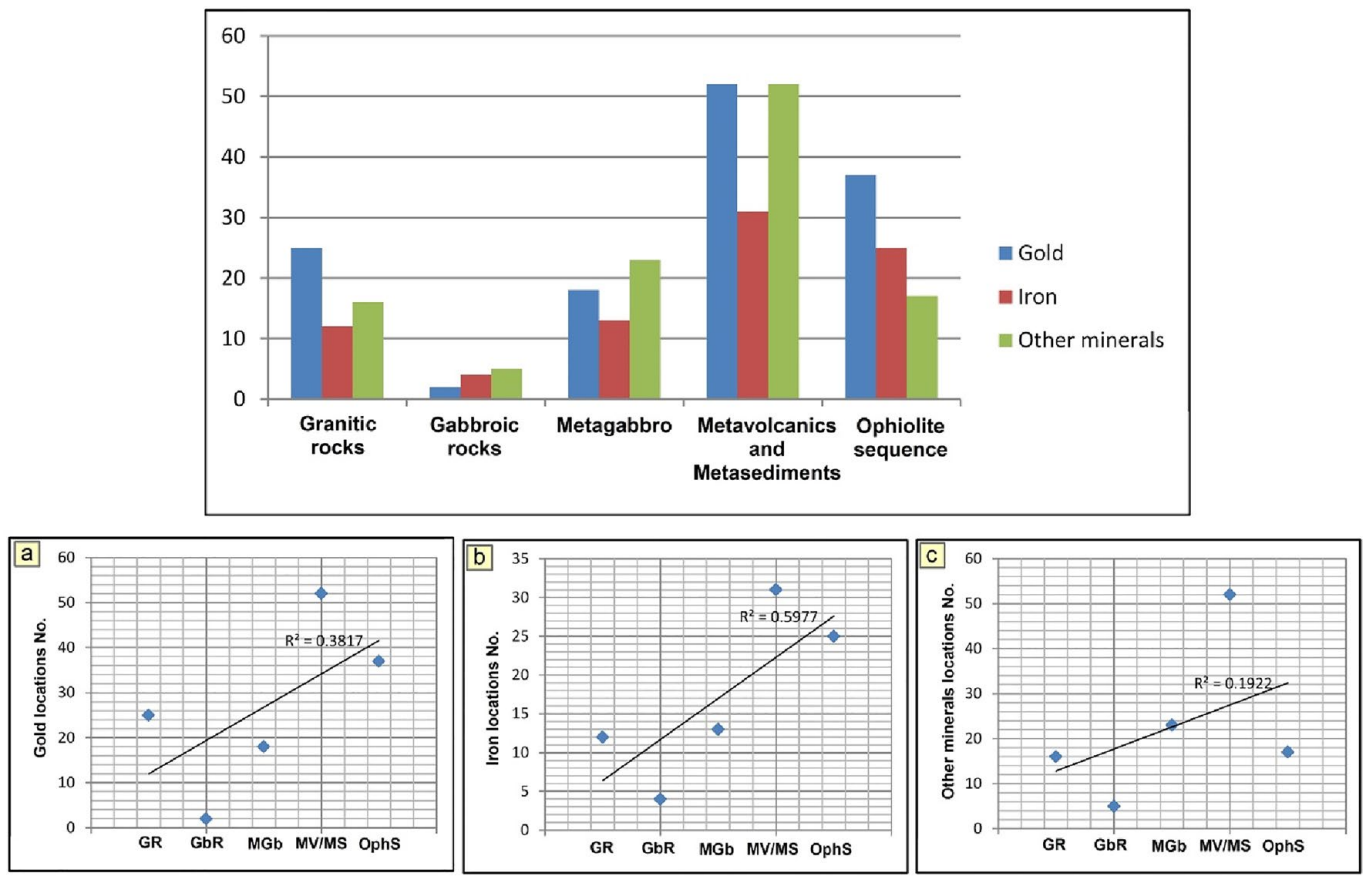
Correlation results show the relation between mineralization localities and five basement rock units (GR: granitic rocks, GbR: gabbroic rocks, MGb: metagabbro, MV/MS: metavolcanics/metasediments, and OphS: ophiolite sequence).

In order to know the relationship between the occurrences of mineralization (gold, iron, and other minerals; Fig. 10) and the exposed basement rocks, a comparison was made between their location and five major lithological units, which are: granitic, gabbroic, metagabbro, metavolcanics/metasediments, and ophiolite sequence (Fig. 10). These units were chosen mainly according to the previous geological and mineralogical information^62,104^. Due to the general agreement between the lithological units’ distributions resulted from PC analysis, MNf transform, and FCC image of Landsat-9 B-Ratio (Figs. 6, 7, 8) with the previous geological map (Fig. 3), the locations of these units were mapped. Statistics were calculated and data correlation values were plotted in a histogram to facilitate figuring out the interrelationship (Fig. 11). This comparison shows the following results:

- In general, places of mineralization of various types are frequently associated with metavolcanics/metasediments, while it appears less closely associated to gabbroic rocks.
- Most of gold minerals localities are hosted in metavolcanics/metasediments (~ 52 localities) then followed by ophiolite sequence and granitic rocks (~ 35 and ~ 25 localities). This is consistent with most previous researches that focused on studying gold mineralization^105,106^. This diversity related to the host rock, fluid/rock interaction, and the tectonic events that cause hydrothermal fluid movement. Whereas the extensive metavolcanics/metasediments host rocks are essential geochemically in the precipitation of gold due to the metamorphic hydrothermal fluids during submarine volcanic activity^107,108^. Contrary, the orogenic gold deposits that are associated with ophiolite sequence are related to the role of granites in melting and replacement minerals along faults during syn-late orogeny stages^109^.
- Iron minerals have a different behavior with the selected rock units. These minerals are found in few places compared to gold deposits, which may be related to their origin^62^. The greatest number of iron minerals localities that match both metavolcanics/metasediments and ophiolite sequence are relatively equal. It is also compatible with both granitic and metagabbroic rocks by low places (Fig. 11).
- Other minerals (see section Geological framework and Mineralogical Data) show an inverse relationship with rock units compared to iron minerals. The largest number of their localities occurred in metavolcanics/metasediments (Fig. 11), and then followed by metagabbroic rocks (~ 23 localities). There is a great convergence in the places of other minerals that are found with granitic and ophiolite sequence rocks. This result is different from preceding investigations that related to uranium and/or rare metals exploration^67,68,110^.

**Figure 12:**
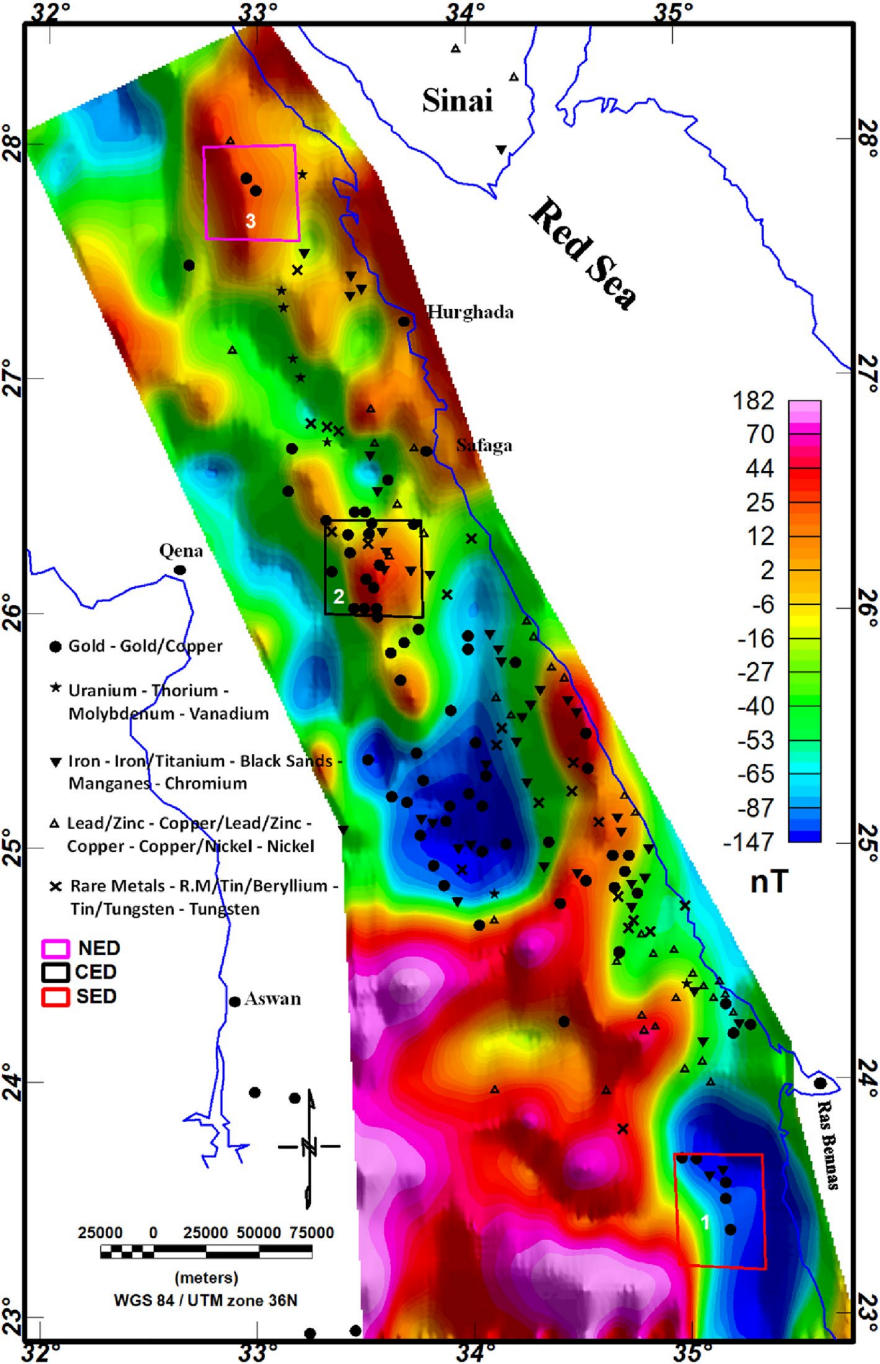
RTP map of the ED.

In this study, we utilized the EMAG2 v3 data. EMAG2 data is a global collection of magnetic data derived from satellite measures. It delivers a smooth representation of magnetic anomaly data across the Earth's surface, with a resolution of about 2 arc minutes. These data are generated through the collection and processing of magnetic measurements gathered by various satellite missions, including Low-Earth-Orbit (LEO) satellites equipped with magnetometers. The EMAG2 v3 data acquisition requires the processing of magnetic field (MF) measurements gathered by LEO. These measurements are collected using fluxgate magnetometers or other magnetometer devices onboard the satellites. These raw magnetic data are gridded, processed and enhanced to produce the final EMAG2 dataset. The EMAG2 v3-magnetic anomaly map (Fig. 5) underwent a transformation to magnetic pole reduction (RTP) proposed by Baranov^111^, resulting in the producing of the RTP map, as illustrated in Fig. 12. This map effectively illustrates magnetic relief variations spanning from − 147 to 182 nT (Fig. 12), showing a spectrum of both positive and negative magnetic responses. Particularly, the southern part of the ED exhibits the highest positive magnetic anomalies, represented by red to pink colors. To establish correlations between mineralization sites and the magnetization of rocks of the ED, the known mineralization sites (Fig. 4) were superimposed to the RTP data and other magnetic maps.

**Figure 13:**
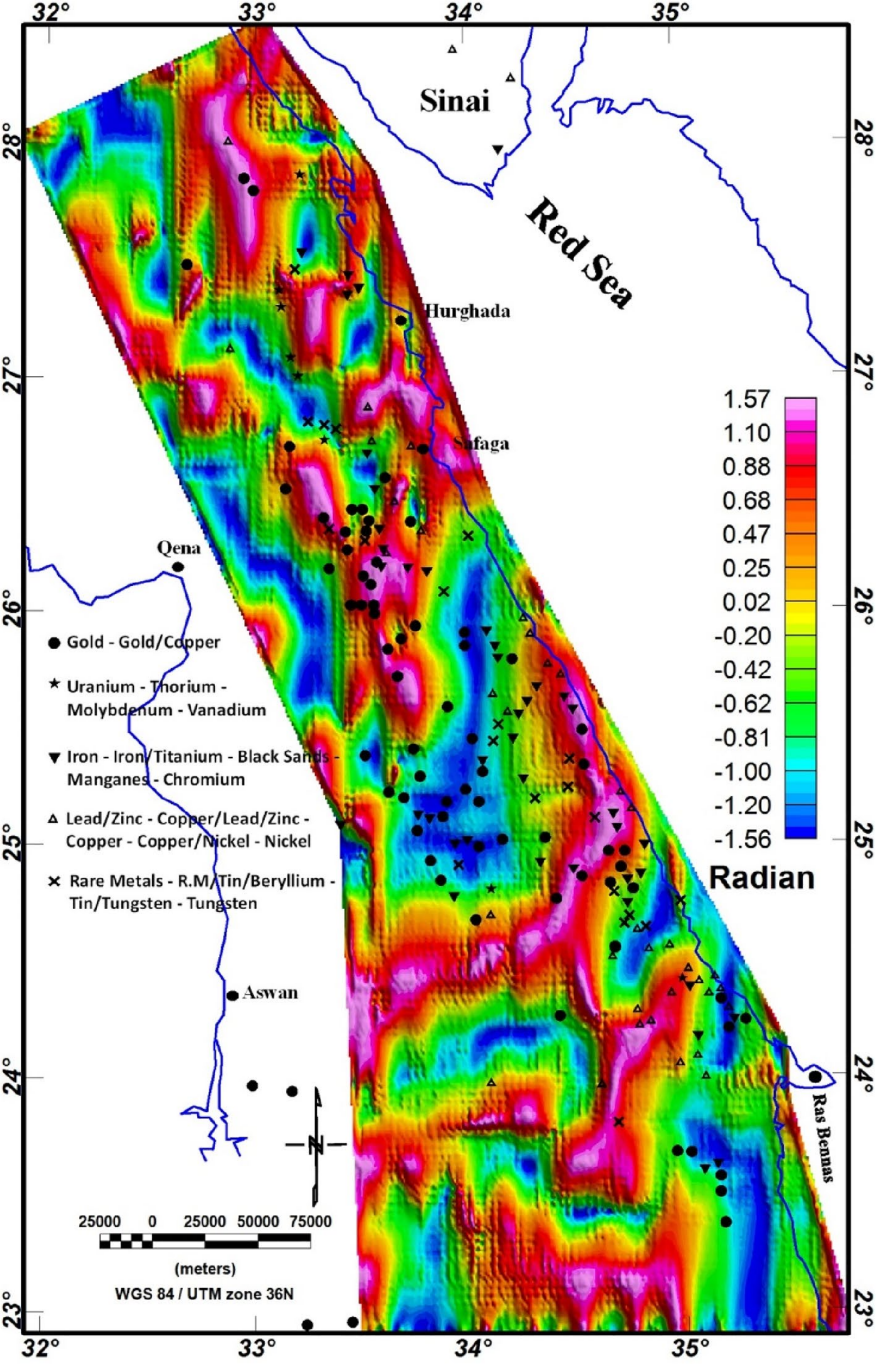
TDR map of the ED.

Upon closer inspection, it becomes evident that the majority of mineralization sites align with negative magnetic anomalies or the boundaries between negative and positive anomalies. For further delineation of structural features, the TDR filter was systematically applied on the RTP map, revealing distinctive lineaments within the ED (Fig. 13). Both the RTP and TDR maps (Figs. 12, 13) provide compelling evidence that the three provinces within the ED—SED, CED, and NED—exhibit discernible separation through major tectonic discontinuities, notably manifested as major shear belts.

**Figure 14:**
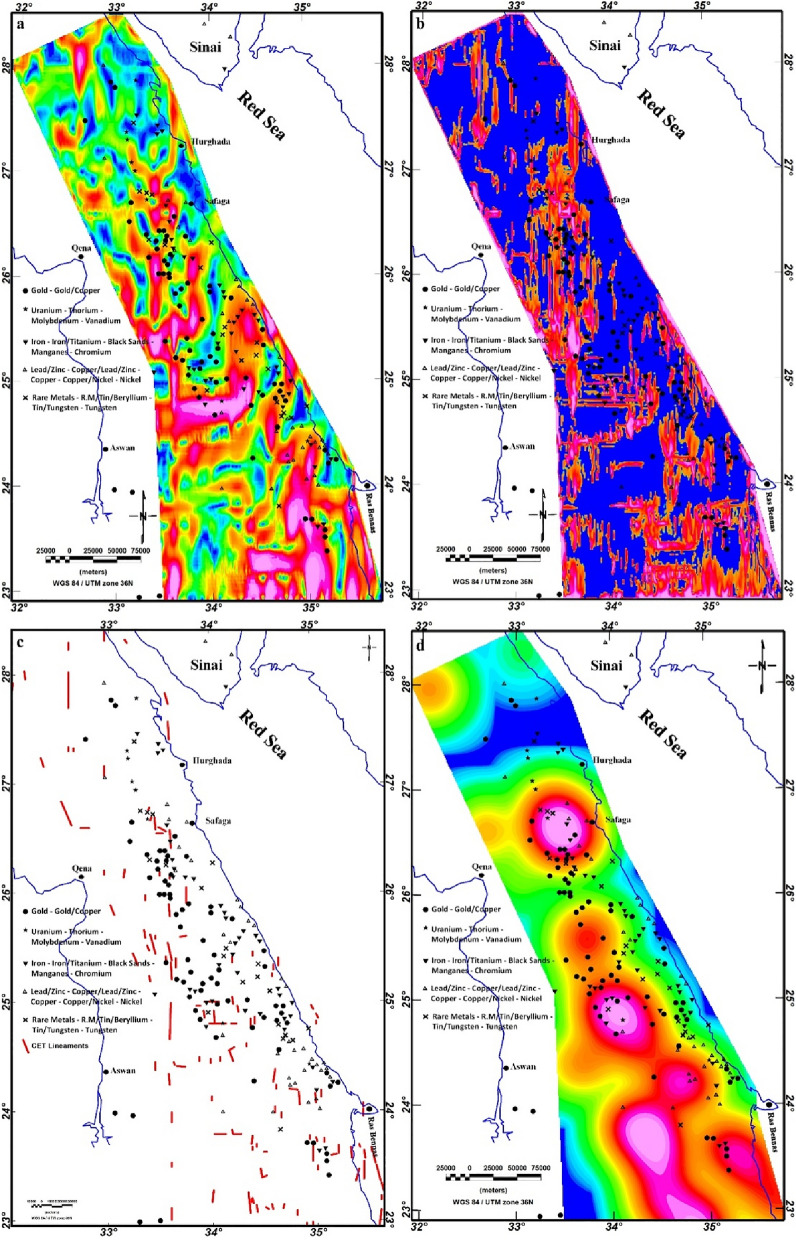
CET-GA map: (**a)** STD map; (**b)** PSE map; (**c**) DS map; and (**d**) COD map of the ED.

The CET-GA method serves as a robust technique for probing the texture of an image, enabling the identification of zones characterized by structural complexity. This technique, depicted in Fig. 14, focuses on assessing the potential occurrence of mineral deposits. The CET-GA initiates by pinpointing magnetic discontinuities, subsequently delineating areas marked by discontinuity. Structural linkages are then scrutinized to unveil junctions, crossings, contacts, and alterations in strike direction. This systematic approach facilitates the selection of promising localities for further investigation^21,22^.

Various maps resulted from this comprehensive analysis, including Standard Deviation (STD), Phase-Symmetry-Edges (PSE), Delineation of Structures (DS), and the Contact-Occurrence-Density Map (COD), also referred to as the heat map. The COD map provides a direct indication for zones of complex structures, serving as a significant guide to probable mineralization locations. The outcomes from this analysis are visually shown in Fig. 14. Through an integrated interpretation of the STD, PSE, and DS maps (depicted in Fig. 14a–c), the COD (heat) map (Fig. 14d) is presented. This heat map specifically addresses the probable mineralization locations, emphasizing strong junctions and fracture regions.

**Figure 15:**
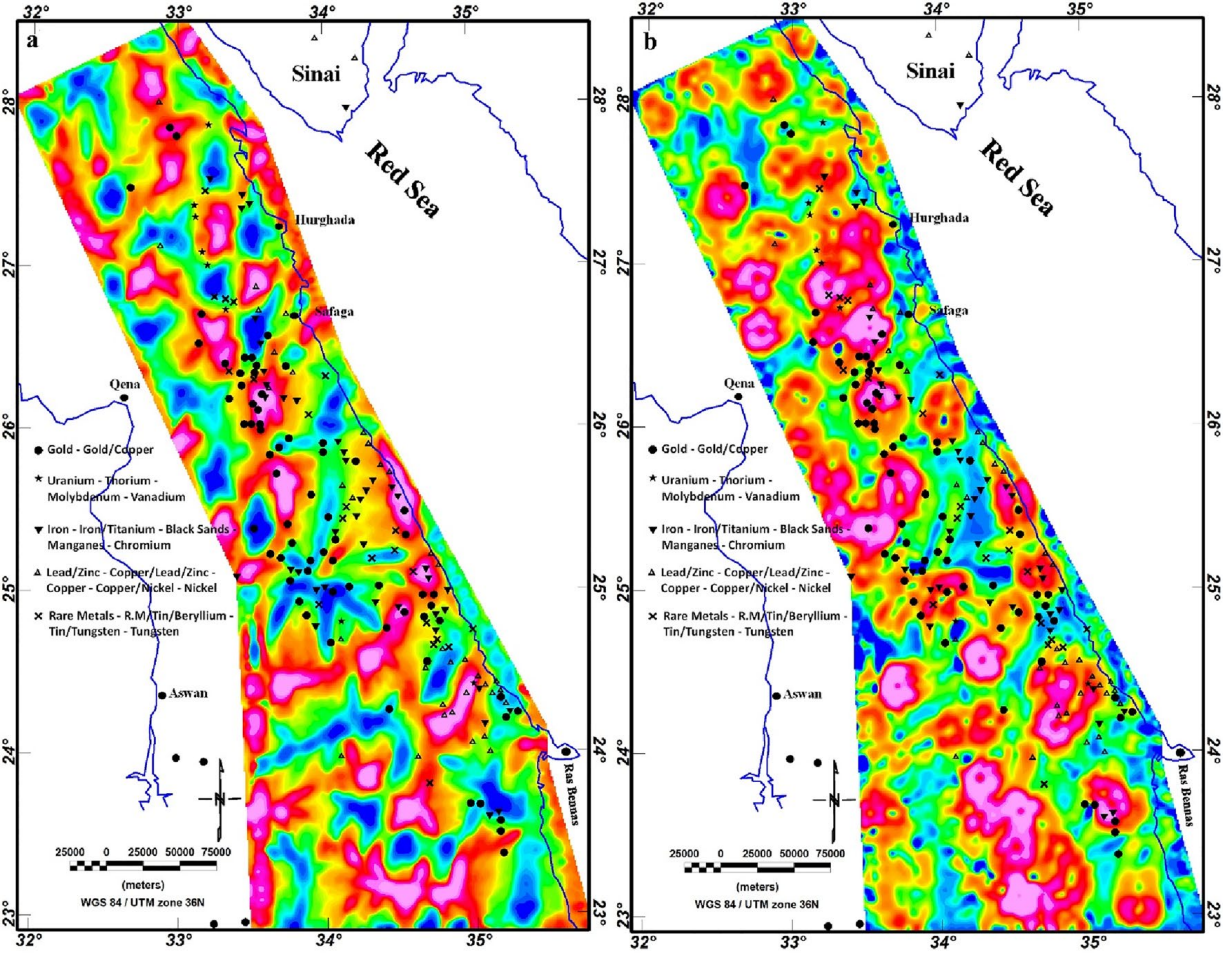
CET-GA maps: (**a**) Circular Feature Transform (CFT); and (**b**) Amplitude Contrast Transform (ACT) of the ED.

In a complementary step, the CET-PA method is applied to the RTP map of the Egyptian Eastern Desert (ED) to generate the CET-PA map, as illustrated in Fig. 15. This map unveils dyke-like structures and porphyry intrusions distributed along deduced trends, including NW, WNW, NE, N-S, and ENE directions. The prevalence of detected porphyry intrusions is notably linked to the basement complexes. Figures 14 and 15 collectively illustrate that a significant number of known mineralization sites are correlated with regions featuring medium to high structural density and porphyry intrusions.

## Discussion

Specifically, the Qena-Safaga Shear Zone is identified between the NED and CED, with the potential presence of the Wadi-Kharit-Wadi Hodein Shear Belt between the CED and SED. While the Idfu-Mersa Alam and Nugrus-Shait Shear Zones are less distinguishable through the interpreted shallow and deep structures, they play a significant role in forming the geological framework. The NED displays extensional structures with dominant E–W and NE–SW tectonic trends. Structural density is relatively low at the surface or near-surface, progressively increasing with depth as indicated by magnetic and gravity data. The age of structures in this part is closely tied to the latest tectonic events affecting the Gondwana lands during the late Ediacaran period (younger than 550 Ma)^99,112–114^.

Moving to the CED, this part is distinguished by a combination of transpressional and extensional structures, kinematically resembling the Najd Orogeny. Predominant tectonic trends include NW–SE and WNW-ESE, with a younger NE-SW trend that intersects the Najd shear trend in certain locations. Lineament density in this area is comparatively lower for both subsurface and near-surface structures. The age of tectonic structures within this extensive region is closely linked to the timing of deformation along the Najd Fault System^114–117^.

Lastly, the SED is characterized by prominent compressional and estimated extrusional structures, with the Allaqi Suture Zone and the Hamisana Shear Zone directly linked to these tectonic movements. Recognized as the oldest and highly deformed structural province, the SED exhibits the highest lineament density among the three provinces. The primary tectonic trends in this region include WNW-to-NW in the western part and N-, NNE-to-NE in the eastern part^99,114,118^.

**Figure 16:**
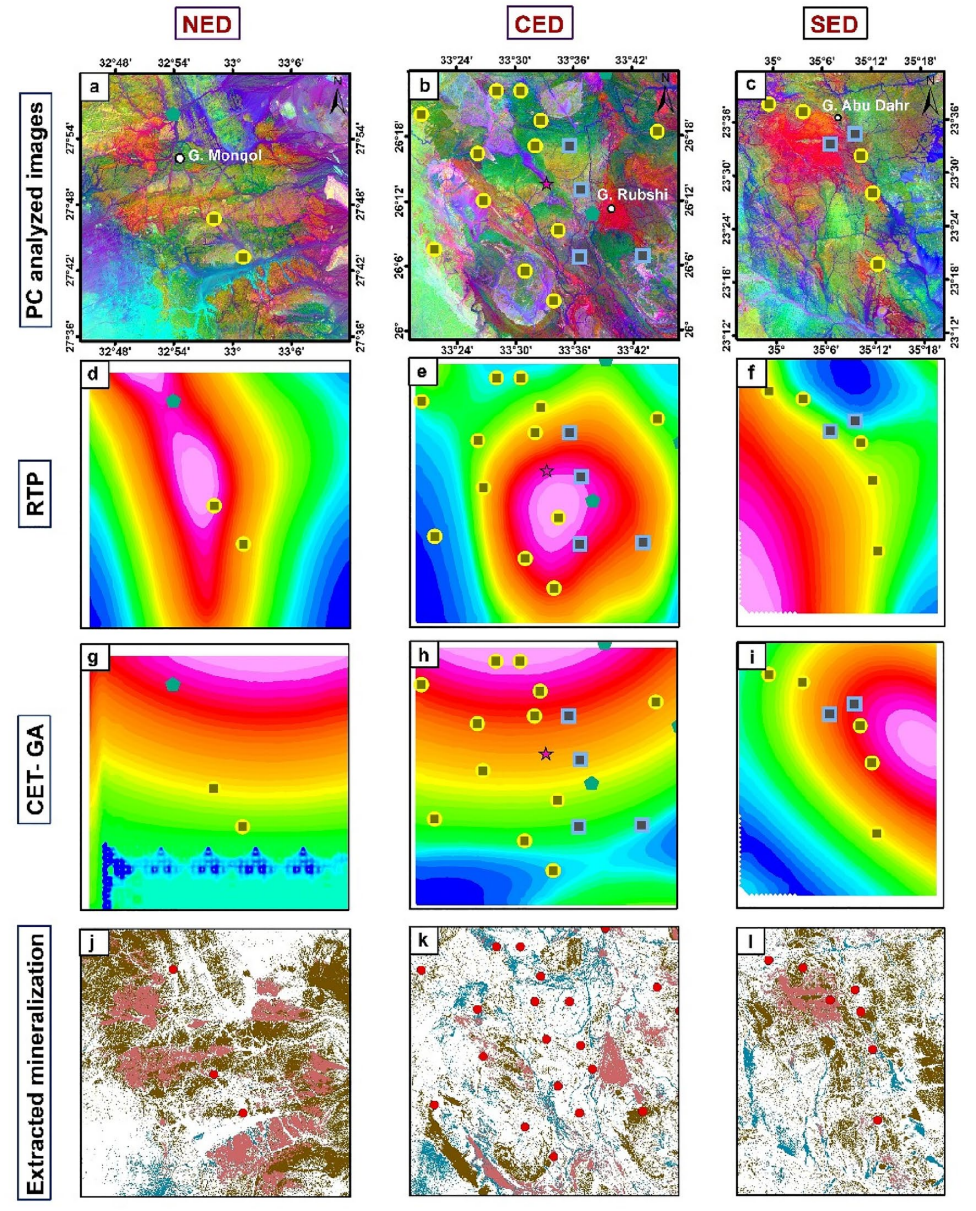
Close-up images for the 3 highlighted areas in Fig. 11: (**a**) PCA-NED; (**b**) PCA-CED; (**c**) PCA-SED; (**d**) RTP-NED; (**e**) RTP-CED; (**f**) RTP-SED; (**g**) CET-GA-NED; (**h**) CET-GA-CED; (**i**) CET-GA-SED; (**j**) Extracted mineralization-NED; (**k**) Extracted mineralization-CED; and (**l**) Extracted mineralization-SED.

For a more detailed understanding, close-up images were showed of three separate regions representing the three main sectors (NED, CED, & SED) of the ED (Highlighted in Fig. 12), in order to confirm and clarify the relationship between known mineralization sites, magnetic responses and geological factors, including rock units and structural features. Generally, these close-up images confirm the previously mentioned results in addition to indicating the association of known mineralization zones with the boundaries between rock units with each other, particularly in NED and SED sectors (Fig. 16). Moreover, most mineralized zones are associated with medium-to-high magnetic sources (Fig. 16d–f) and with high-structural-complexity zones (Fig. 16g–i). This reveals the important role of main tectonic events in forming these minerals. On the other hand, in CED part, it was noticed that mineralization is widespread, not only in the boundaries between lithological units, but also within their masses themselves. This shows that the rocks were severely affected by multiple intensive structural events. The comparison between the documented mineralization sites with the resultant extracted ones reveals that the regions in which the three altered indices are present are the ones that are interested and have priority for exploration (Fig. 16). This clear visualization underscores the robust relationship between the intricate structural fabric and mineralization within the Egyptian ED. The strength of this correlation improved our understanding of the governing geological dynamics for mineralization processes in this region, contributing valuable insights to the broader field for exploring mineralization.

## Conclusion

This extensive study reveals the invaluable integration of Landsat-9 images and space geophysical magnetic data in delineating essential factors for mineralization exploration in the Egyptian Eastern Desert (ED). The analysis, combining advanced approaches such as Principal Component (PC) analysis and Minimum Noise Fraction (MNf) transform, provides precise lithological mapping, highlighting the significance of employing multiple analysis methods for enhanced geological accurateness. The composite images, particularly those emanating from specific PC and MNf bands; demonstrate superior in identifying lithological units. The study underlines distinct lithological compositions across the ED, showing the abundance of ophiolite sequences and metamorphic rocks in the south (SED), and the prevalence of granitic rocks and Hammamat clastics in the north (NED). On the other hand, the central part (CED) of ED is distinguished by a great variety of existing rock units. Noteworthy is the challenge in discriminating Hammamat clastics, attributed to their small spatial distribution and close composition parallel to other lithological units. Despite this, topography assists in accurate differentiation, particularly for granitic rocks.

The study underscores the effectiveness of applied research methods in classifying basement rocks across the extended ED, aligning with common lithological classifications. This variety in basement rocks, marked by variations in presence, origin, and tectonic events, arises as a key driver for the various mineral occurrences in the region. Hydrothermal alteration mapping, facilitated by B-Ratio results and false color composite images, confirms that it is instrumental in recognizing preliminary areas for mineral discovery. Discrimination of altered zones and mapping of iron oxides, ferrous silicates, and clay/OH group minerals through specific B-Ratio further enhances the interpretation of mineralization processes. The results, distinct from weathering-related patterns, display high precision in pinpointing sites of alteration and mineralization. Correlation analyses between mineralization styles and major lithological units reveal significant associations. Particularly, metavolcanics/metasediments appear as frequent hosts for various mineral types, while gabbroic rocks show less frequent associations. Gold mineralization, concentrated in metavolcanics/metasediments, aligns with the previous studies, showcasing consistency in findings due to the availability of multiple factors that cause gold occurrences such as comprehensive deformation events and the released hydrothermal fluids accompanying them. Iron minerals, appearing less frequently than gold, exhibit relatively equal relationships with metavolcanics/metasediments and ophiolite sequence, which is due to the nature of their composition that is defined as syn-depositional within layered volcanic-volcaniclastic sequences. Other minerals, showing an inverse relationship with iron minerals, notably concentrate in metavolcanics/metasediments and metagabbroic rocks.

The examination of the EMAG2 v3-magnetic anomaly map, converted through magnetic pole reduction (RTP), highlights distinguishable magnetic responses across the ED. The correlation of mineralization locations with magnetic anomalies and the delineation of tectonic structures through the Tilt Derivative of RTP (TDR) maps reveal noticeable separations among the three ED provinces (SED, CED, NED) through major tectonic discontinuities. Structural elements, further highlighted by the Center for Exploration-Targeting Grid-Analysis (CET-GA) with Center for Exploration-Targeting Porphyry-Analysis (CET-GA) approaches, underscore zones of complicated structures with medium to high structural density and porphyry intrusions. The correlation of these features with mineralization sites represents a strong relationship, providing a valuable understanding of the geological dynamics controlling mineralization processes.

In conclusion, this research significantly promotes exploration in the Egyptian ED, suggesting an advanced understanding of lithological variations, hydrothermal alteration processes, and structural complexities. The integration of different datasets, progressive analysis methods, and correlation investigations contribute to a comprehensive framework for mineralization exploration, assisting in the detection of prospective zones and improving our experience of geological settings.

## Data Availability

Data sets generated during the current study are available from the corresponding author on reasonable request.
